# Theranostic molecular profiling of pleomorphic ductal carcinoma of the breast

**DOI:** 10.1111/tbj.13187

**Published:** 2018-12-18

**Authors:** Semir Vranic, Juan Palazzo, Jeffrey Swensen, Joanne Xiu, Elena Florento, Zoran Gatalica

**Affiliations:** ^1^ Department of Pathology, Clinical Center University of Sarajevo Sarajevo Bosnia and Herzegovina; ^2^ School of Medicine University of Sarajevo Sarajevo Bosnia and Herzegovina; ^3^ College of Medicine Qatar University Doha Qatar; ^4^ Department of Pathology, Anatomy and Cell Biology Thomas Jefferson University Hospital Philadelphia Pennsylvania; ^5^ Caris Life Sciences Phoenix Arizona

**Figure 1:**
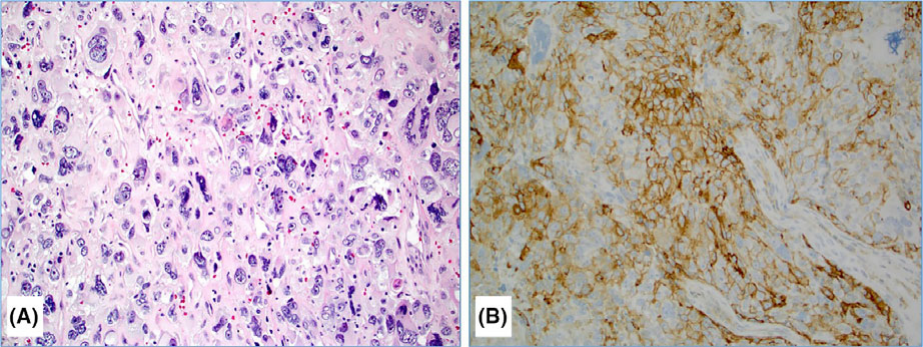
A case of pleomorphic ductal carcinoma with a diffuse infiltration of highly pleomorphic neoplastic cells some of which with a bizarre and multinuclear appearance (Hematoxylin & Eosin stain, 20×) (A); this was the only pleomorphic ductal carcinoma with a significant (20% of tumor cells) PD‐L1 expression (20×) (B) [Color figure can be viewed at wileyonlinelibrary.com]

Pleomorphic ductal carcinoma (PDC) is a very rare subtype of invasive ductal carcinoma of no‐special type (NST), characterized by the presence of highly atypical/bizarre (>6‐fold variation in nuclear size) and multinucleated (giant) neoplastic cells comprising >50% of the tumor cell population^1^ (Figure 1A). PDC is typically triple‐negative breast cancer (TNBC), associated with an aggressive clinical course and a poor outcome.^2^, ^3^, ^4^ So far, no single study explored novel predictive biomarkers for the precision medicine purposes in the patients with PDC. Formalin‐fixed paraffin‐embedded tissue samples of the six PDC patients (four primary and two metastatic cases) were sequenced for 592‐genes using NextSeq platform (Illumina, La Jolla, CA, USA). Tumor mutational load (TML) was calculated using only somatic nonsynonymous missense mutations; high TML was considered when it was ≥17 mutations/Mb. Microsatellite instability (MSI) status was explored by the direct analysis of known MSI loci in the target regions of the sequenced genes. Cases were considered microsatellite instable (MSI‐H) if they exhibited ≥46 altered microsatellite loci (the threshold was established by comparing to the PCR‐based MSI FA result from ~2100 cases^5^). Copy number variations (CNVs) were determined by comparing the depth of sequencing of genomic loci with a diploid control. Calculated gains ≥6 copies were considered amplified. ArcherDx FusionPlex Assay was used to detect gene fusions (52 gene targets). Immunohistochemistry was used to detect expression of PD‐L1 (SP142 antibody, Ventana) in tumor cells (TC) and immune cells (IC). PD‐L1 positivity in TC was defined as 2+ intensity in ≥5% of tumor cells.^6^ PD‐L1 status in ICs was categorized as IC0 (<1%), IC1 (≥1% but <5%), and IC2/3 (≥5%).^7^ All tests were performed at Caris Life Sciences (Phoenix, AZ), and details are available at https://www.carismolecularintelligence.com/tumor-profiling-menu/mi-profile-usa-excluding-new-york/).

All PDCs were confirmed to be of the breast origin and positive for epithelial markers (eg, AE1/AE3, Cam5.2). Estrogen receptor (ER), progesterone receptor (PR), and Her‐2/neu protein were negative in all cases. *TP53* mutations were detected in five of six cases, with one case harboring two additional pathogenic mutations (*SMARCA4* R1093X and *Fumarate Hydratase* K477dup), and two cases with pathogenic *BRCA1* (E143X) or *KRAS* (G12A) mutations (Table 1). No pathogenic mutation was detected in one case. No gene fusions were detected in any of the cases successfully analyzed (0/4). Gene amplification of cyclin‐dependent kinase inhibitor 1B (*CDKN1B*) and fibroblast growth factor receptor 1 (*FGFR1*) genes was detected in one case, each. These results indicate that PDCs exhibit significantly less targetable genetic alterations in contrast to related TNBC and metaplastic breast carcinomas.^8^, ^9^ A single case with a mutation in *BRCA1* gene indicates a potential benefit to platinum compounds and PARP inhibitors.^10^ Tumor expression of PD‐L1 (TC) was negative/low in all but one case that exhibited 20% positive tumor cell population (Figure 1B), while IC expressing PD‐L1 were detected at potentially significant levels (IC2; ≥5%<50%) in three cases. Total mutational load (TML) was low in all cases (range, 4‐11/Mb), and no DNA microsatellite instability was detected in any case (all five cases were microsatellite stable) (Table 1). Low TML, rare PD‐L1 expression (1/6 TC+ and 3/5 IC+), and absence of mismatch repair deficiency make this tumor an inconsistent candidate for treatment with the current immune checkpoint inhibitors.^5^ We encourage further studies on PDC to reveal novel predictive biomarkers for this rare and difficult‐to‐treat breast cancer subtype.

**Table 1:** A summary of the molecular profiling results on six pleomorphic ductal carcinomas of the breast

PDC	TML	MSI	*BRCA1*	*BRCA2*	*TP53*	Other NGS	PD‐L1 (TC)	PD‐L1 (IC)	CNV	Archer
Case#1 (primary)	6/Mb	Stable	E143X	wt	R273C	None	20% (2+ intensity)	IC2	None	None
Case#2 (primary)	11/Mb	Stable	wt	wt	R342P	*KRAS* G12A	1% (2+ intensity)	N/E (necrosis)	*CDKN1B↑*	None
Case#3 (primary)	4/Mb	Stable	wt	wt	H193R	None	Negative	IC1	None	None
Case#4 (primary)	4/Mb	Stable	wt	wt	E294fs	VUS	Negative	IC2	*FGFR1↑*	Failed
Case#5 (metastatic)	7/Mb	Stable	wt	wt	wt	SNPs	3% (2+ intensity)	IC2	None	None
Case#6 (metastatic)	7/Mb	Stable	wt	wt	R248Q	*SMARCA4* *FH*	Negative	Negative	None	Failed

*CDKN1B*, cyclin‐dependent kinase inhibitor 1B; CNV, copy number variations; *FGFR1,* fibroblast growth factor receptor 1; *FH*, fumarate hydratase gene; IC, immune cells; Mb, megabase; MSI, microsatellite instability; N/E, not evaluated; NGS, next‐generation sequencing; PDC, pleomorphic ductal carcinoma; SNP, single‐nucleotide polymorphism; TC, tumor cells; TML, tumor mutational load; VUS, variant of unknown significance; wt, wild type; ↑: amplified.
